# Adsorption of DNA nucleobases on single-layer Ti_3_C_2_ MXene and graphene: vdW-corrected DFT and NEGF studies

**DOI:** 10.1063/5.0160784

**Published:** 2023-08-08

**Authors:** Benjamin O. Tayo, Michael A. Walkup, Serkan Caliskan

**Affiliations:** 1 School of Engineering, University of Central Oklahoma, Edmond, Oklahoma 73034, USA; 2 Department of Physical and Applied Sciences, University of Houston–Clear Lake, Houston, Texas 77058, USA

## Abstract

We investigated the interaction of DNA nucleobases [adenine (A), guanine (G), thymine (T), and cytosine (C)] with single-layer Ti_3_C_2_ MXene using Van der Waals (vdW)-corrected density functional theory and non-equilibrium Green’s function methods. All calculations were benchmarked against graphene. We showed that depending on the initial vertical height of a nucleobase above the Ti_3_C_2_ surface, two interaction mechanisms are possible, namely, physisorption and chemisorption. For graphene, DNA nucleobases always physisorbed onto the graphene surface irrespective of the initial vertical height of the nucleobase above the graphene sheet. The PBE+vdW binding energies for graphene are high (0.55–0.74 eV) and follow the order G > A > T > C, with adsorption heights in the range of 3.16–3.22 Å, indicating strong physisorption. For Ti_3_C_2_, the PBE+vdW binding energies are relatively weaker (0.16–0.20 eV) and follow the order A > G = T > C, with adsorption heights in the range of 5.51–5.60 Å, indicating weak physisorption. The binding energies for chemisorption follow the order G > A > T > C, which is the same order for physisorption. The binding energy values (5.3–7.5 eV) indicate very strong chemisorption (∼40 times larger than the physisorption binding energies). Furthermore, our band structure and electronic transport analysis showed that for physisorption, there is neither significant variation in the band structure nor modulation in the transmission function and device density of states. The relatively weak physisorption and strong chemisorption show that Ti_3_C_2_ might not be capable of identifying DNA nucleobases using the physisorption method.

## I. INTRODUCTION

The interaction of DNA nucleobases with atomically thin two-dimensional (2D) materials has garnered significant interest in the field of materials science because of its importance in single-molecule detection and DNA sequencing. Indeed, a great number of theoretical and experimental studies have been performed to investigate the viability of various 2D materials for DNA sequencing, such as graphene,^1,2^ hexagonal boron nitride (hBN),^2–5^ molybdenum disulfide (MoS_2_),^5–8^ tungsten disulfide (WS_2_),^9^ phosphorene,^10–12^ silicene,^12^ and borophene,^13^ with varying levels of success and challenges. For instance, the single-layer nature of graphene makes it ideal for detecting DNA nucleobases at the single-base level. However, due to graphene’s nonpolar surface, DNA nucleobases interact with graphene via *π*–*π* interaction, which produces strong physisorption of the nucleobases.^2^ This strong physisorption can cause nucleobases to stick to the surface of graphene, slowing down the translocation rate and increasing error rates as multiple bases can interact with graphene’s surface at any given time.^14,15^ hBN exhibits the same honeycomb lattice structure as graphene; however, it is a polar insulator, while graphene is a non-polar gapless semimetal.^2,3^ Due to its polar nature, hBN sheets can produce spatial resolutions for DNA sequencing slightly better than graphene.^3^ Similar to hBN, MoS_2_ membranes are polar, direct bandgap semiconductors and have been shown to have better ability in detecting the individual nucleobases in terms of a higher signal-to-noise ratio and have lower tendency of nucleobases to stick to its surface.^6^ However, MoS_2_ membranes can suffer degradation over time when repeatedly exposed to high electric fields. Recently, 2D MXenes have emerged as promising alternative materials for DNA nucleobase detection. MXenes have the general formula *M*_*n*+1_*X*_*n*_T_*x*_, where *M* is a transition metal (such as Ti, V, Nb, and Mo), X is carbon or nitrogen, T_*x*_ represents different functional groups (such as –O, –F, and –OH) on the MXene surface, and *n* can take values between 1 and 3.^16^ Molecular dynamics simulation studies using Ti_3_C_2_ MXene nanopores by Yadev *et al.*^17^ and Cao *et al.*^18^ showed their potential in detecting nucleobases based on physical features such as ionic current and dwell time. Another study on 2D Ti_2_C(OH)_2_ MXene nanopores combining density functional theory (DFT) and the non-equilibrium Green’s function (NEGF) method by Prasongkit *et al.*^19^ showed that nucleobases can be detected using the transverse conductance spectrum. Furthermore, combining DFT, NEGF, and supervised machine learning techniques, Mittal *et al.*^20^ demonstrated the possibility of detecting both DNA and methylated DNA nucleobases using a Ti_2_NS_2_ MXene nanochannel device.

So far, most of the studies for DNA sequencing using 2D MXenes have been based on the nanopore^18,19^ or nanochannel^20^ method. Another useful detection mechanism for DNA nucleobases is the physisorption mechanism, where the changes in sheet current due to physisorption of DNA nucleobases can be measured.^1,2^ In this work, we study the adsorption of DNA nucleobases on 2D Ti_3_C_2_ MXene using the Van der Waals (vdW)-corrected DFT and NEGF technique. The binding energy of nucleobases on the Ti_3_C_2_ sheet was benchmarked against graphene. We find that for graphene, the DNA nucleobases physisorbed strongly on the graphene sheet irrespective of the initial vertical height between the nucleobase and the graphene surface. For the Ti_3_C_2_ sheet, two interaction mechanisms were observed. Depending on the initial vertical height of DNA bases above the Ti_3_C_2_ sheet, the final equilibrium geometry showed that nucleobases interact with the Ti_3_C_2_ sheet either via physisorption (relatively weak compared to graphene) or chemisorption. Furthermore, our band structure and electronic transport analysis showed that for physisorption, there is neither significant variation in the band structure nor modulation in the transmission function and the device density of states (DDOS). The relatively weak physisorption and strong chemisorption show that Ti_3_C_2_ might not be capable of identifying DNA nucleobases using the physisorption method.

## II. COMPUTATIONAL METHODS

We performed relaxation calculations separately for the 2D membranes (Ti_3_C_2_ MXene and graphene) and the DNA nucleobases. For the nucleobases, each nucleobase was placed in a large simulation box of a dimension of 15 × 15 × 30 Å^3^ to avoid spurious interaction between the nucleobase and its periodic image. For graphene and Ti_3_C_2_, the unit cells were first optimized; then using the optimized coordinates, a 5 × 5 supercell was created, which is large enough for adsorption studies of nucleobases.^2^ For the relaxation calculations for graphene and Ti_3_C_2_, a vacuum of 30 Å was used to avoid interlayer interactions. For adsorption studies, each nucleobase was placed approximately above the center of the 5 × 5 supercells for Ti_3_C_2_ and graphene. All relaxation calculations were performed using DFT. For DFT studies, we modeled the exchange-correlation potential within the generalized gradient approximation (GGA) using the Perdew–Burke–Ernzerhof (PBE) functional.^21^ Van der Waals corrections were included using the Grimme-D2 functional.^22^ All the relaxation calculations were performed using a 1 × 1 × 1 k-space integration grid, a wave function cut-off of 45 Ry, and a charge density cut-off of 450 Ry. All atoms were relaxed until the residual forces between atoms were less than 0.01 eV/Å. The band structure was calculated for each system using a denser k-space integration grid of 5 × 5 × 1. All DFT calculations were performed using the Quantum ESPRESSO software.^23,24^ The transmission function was computed using the NEGF method. The NEGF calculations were performed using the QuantumATK software.^25^ Computational resources were provided by the University of Central Oklahoma’s Buddy Supercomputer Center.^26^

## III. RESULTS

### A. DNA nucleobases

The energy gap (*E*_*g*_) of the nucleobases A (adenine), G (Guanine), C (Cytosine), and T (Thymine) was calculated as the difference between the LOMO (lowest occupied molecular orbital) and HOMO (highest occupied molecular orbital). Table I shows the energy gaps of the nucleobases. It is observed that the PBE and PBE+vdW corrected energy gaps are almost identical and comparable with those obtained from previous studies.^2,6,27^

**TABLE I.** HOMO–LOMO gaps of DNA nucleobases.

Base	PBE *E_g_* (eV)	PBE+vdW *E_g_* (eV)
A	3.838	3.836
G	3.467	3.465
T	3.760	3.757
C	3.670	3.669

### B. Pristine Ti_3_C_2_ and graphene

**FIG. 1.**
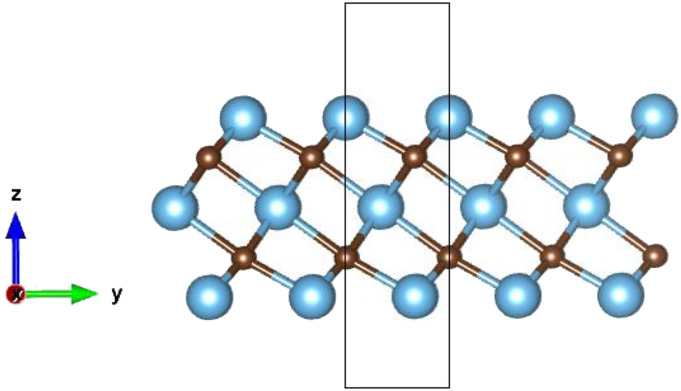
Side view of the optimized unit cell of Ti_3_C_2_ obtained with the PBE+vdW method. Blue circle: Ti atom; brown circle: C-atom.

Ti_3_C_2_ MXene is a 2D hexagonal system that belongs to the P63/mmc space group. The unit cell of Ti_3_C_3_ is shown in Fig. 1. Ti_3_C_2_’s crystal structure consists of five planes. Planes 1, 3, and 5 are made of Ti atoms, with the C atoms in planes 2 and 4 sandwiched between the Ti planes. The optimized bond lengths and lattice constant are given in Table II. The Ti–C–Ti bond angle was 97.8° (PBE) and 97.5° (PBE+vdW). The geometric parameters are consistent with values reported in other studies.^28,29^

**TABLE II.** Optimized parameters (in Å) for Ti_3_C_2_ and graphene unit cells.

	Parameter	PBE	PBE+vdW
Ti_3_C_2_	Ti–C bond length	2.049	2.052
	C–Ti bond length	2.215	2.212
	Ti–C bond length	2.049	2.052
	Lattice constant	3.088	3.084
Graphene	C–C bond length	1.424	1.424
	Lattice constant	2.465	2.465

**FIG. 2.**
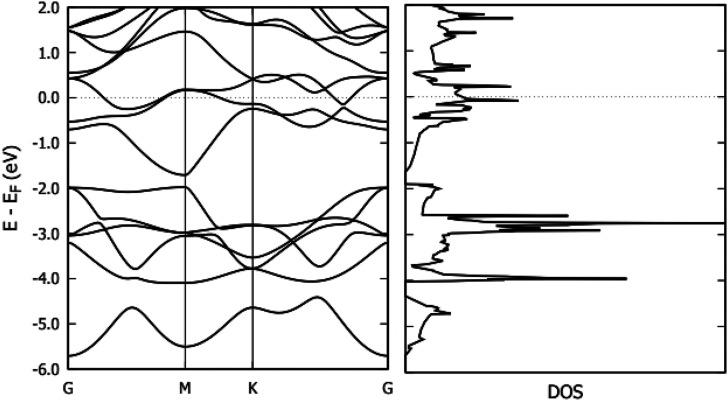
Band structure and DOS of Ti_3_C_2_ using the PBE+vdW method.

Graphene is a 2D hexagonal system consisting of a single plane of carbon atoms. The optimized lattice parameters for the graphene unit cell are shown in Table II. These values are consistent with previous results.^2^ For both PBE and PBE+vdW methods, the optimized C–C–C bond angle was determined to be 120°. The band structure and the Density of States (DOS) for Ti_3_C_2_ are illustrated in Fig. 2. It shows that unlike graphene, which is a zero-band gap semimetal,^30^ single-layer Ti_3_C_2_ exhibits metallic behavior.^29^

### C. Adsorption of DNA nucleobases on graphene and Ti_3_C_2_

The adsorption of DNA nucleobases was carried out by placing the bases above the graphene or Ti_3_C_2_ sheets and performing relaxation calculations to obtain the equilibrium geometry of the system. The binding energy (*E*_*b*_) of a DNA base was determined using the following equation:

$$\begin{array}{c} E_{b}=E_{\mathrm{sub+base}}-E_{\mathrm{sub}}-E_{\mathrm{base}}, \end{array}$$ (1)

where *E*_sub+base_ is total energy for the combined system (Ti_3_C_2_ or graphene substrate + base), *E*_sub_ is the total energy of the substrate (Ti_3_C_2_ or graphene), and *E*_base_ is the total energy of the nucleobase.

**FIG. 3.**
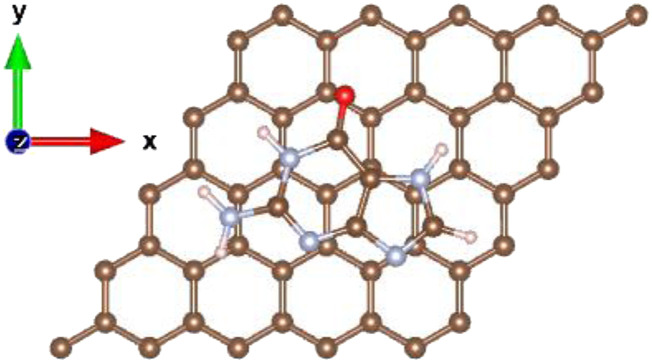
AB stacking configuration of guanine over the 5 × 5 graphene supercell. Carbon is shown in brown, nitrogen in gray, oxygen in red, and hydrogen in pink.

For graphene, optimization calculations were performed in the AB stacking configuration, as shown in Fig. 3. This configuration was determined in previous studies as the energetically favored configuration.^2^ For the optimization calculations for graphene, the DNA nucleobases were placed at different vertical heights in the range from 1.2 to 2.5 Å. Irrespective of the initial vertical height, the DNA nucleobases always physisorbed onto the graphene sheet.^2^ No chemisorption interaction was observed.

**FIG. 4.**
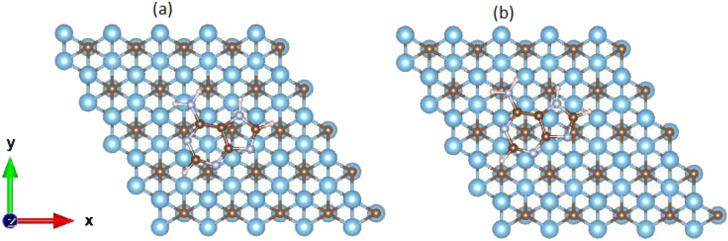
Top view of the stacking configurations of adenine over the 5 × 5 Ti_3_C_2_ supercell: (a) titanium-centered and (b) carbon-centered.

The adsorption of DNA nucleobases on the Ti_3_C_2_ surface was performed for two adsorption sites, that is, the hollow and bridge configurations, as shown in Fig. 4. In the titanium-centered (hollow) configuration, the hexagonal ring in the DNA base is stacked over the hexagonal ring of the Ti_3_C_2_, and the titanium atom on the third layer appears at the center of the ring. In the carbon-centered (bridge) configuration, the hexagonal ring on the DNA base is placed above the titanium-carbon bond so that the carbon atom in the second layer appears at the center of the hexagonal ring on the DNA nucleobase. Relaxation calculations were performed for the two different configurations, and the relative energies were obtained for each nucleobase. After comparing the relative energies for the two configurations, it was determined that the carbon-centered configuration was slightly energetically favorable than the titanium-centered configuration (the difference in energy is less than 1 meV).

**FIG. 5.**
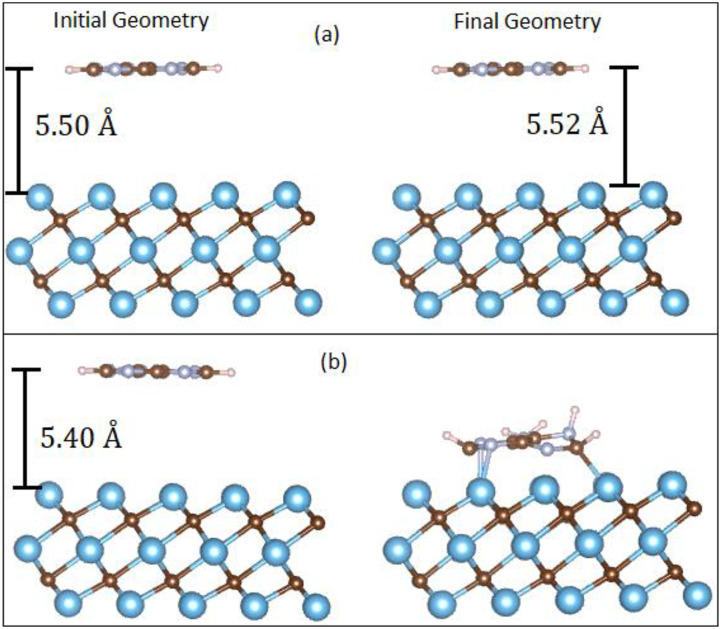
Initial and final geometries (using the PBE+vdW method) of the adenine molecule for different initial vertical heights, illustrating (a) physisorption and (b) chemisorption interaction mechanisms.

Unlike graphene, where the DNA nucleobases only physisorbed onto the graphene sheet, we found that two interaction mechanisms are possible for Ti_3_C_2_ (see Fig. 5). Depending on the initial vertical height of the DNA nucleobase, the final relaxed geometry shows that the nucleobase either physisorbed or chemisorbed on the Ti_3_C_2_ surface. Table III shows the binding energies and interaction mechanisms for different vertical heights of adenine above the Ti_3_C_2_ substrate. From Table III, we observe that when the initial vertical height was less than 5.5 Å, the adenine molecule chemisorbed onto the Ti_3_C_2_ surface, reacting with the surface with very strong binding energies of ∼6 eV. At vertical heights of 5.5 Å and above, the adenine molecule physisorbed onto the Ti_3_C_2_ surface. A similar result was obtained for all the four nucleobases. As discussed in Sec. III C 1, the minimum vertical height for physisorption between the nucleobase and Ti_3_C_2_ depends strongly on the DFT approximation used (PBE or PBE+vdW).

**TABLE III.** Binding energies of adenine adsorbed on the Ti_3_C_2_ surface using the PBE+vdW method.

*d* (Å)	*E*_*b*_ (eV)	Interaction mechanism
5.0	−6.329	Chemisorption
5.1	−7.305	Chemisorption
5.2	−5.524	Chemisorption
5.3	−6.727	Chemisorption
5.4	−6.307	Chemisorption
5.5	−0.195	Physisorption
5.6	−0.183	Physisorption
5.7	−0.171	Physisorption
5.8	−0.160	Physisorption
5.9	−0.150	Physisorption
6.0	−0.141	Physisorption

#### 1. Physisorption of DNA nucleobases on graphene and Ti_3_C_2_

**FIG. 6.**
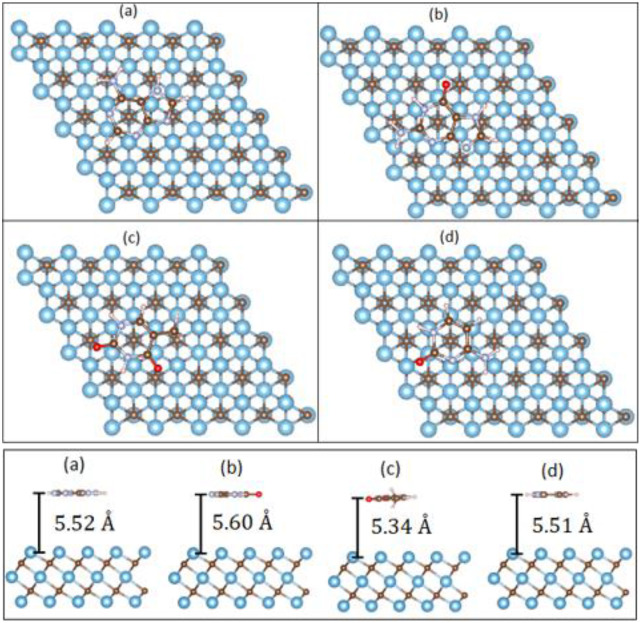
Top view and side view of equilibrium geometries of DNA nucleobases (a) A, (b) G, (c) T, and (d) C physisorbed on the Ti_3_C_2_ surface.

Table IV shows the calculated vertical heights for all four nucleobases physisorbed on graphene or Ti_3_C_2_. Table V lists the binding energies for the four nucleobases. For graphene, the PBE+vdW binding energies are relatively stronger (0.55–0.74 eV) and follow the order G > A > T > C, with adsorption heights in the range of 3.16–3.22 Å, indicating strong physisorption.^2,14,15,31^ This can be attributed to the strong π–π interactions between the nucleobases and graphene.^2^ We note that the PBE vertical heights are larger (3.74–3.77 Å) with smaller binding energies (0.06–0.10 eV). As for Ti_3_C_2_, the PBE+vdW binding energies are relatively weaker (0.16–0.20 eV) and follow the order A > G = T > C, with adsorption heights in the range of 5.51–5.60 Å, indicating weak physisorption. The small binding energies for nucleobases on the Ti_3_C_2_ surface suggest minimal sticking of bases on the Ti_3_C_2_ surface compared to that on graphene.^1,14,15^ The PBE vertical heights are smaller (4.25–4.91 Å), and the binding energies (0.11–0.17 eV) are slightly smaller than the PBE+vdW values. Figure 6 depicts the equilibrium geometries of the DNA bases physisorbed on Ti_3_C_2_.

**TABLE IV.** Calculated vertical height (in Å) between DNA nucleobases and graphene or Ti_3_C_2_.

		Reference	A	G	T	C
Ti_3_C_2_	PBE	This work	4.52	4.63	4.25	4.91
	PBE+vdW	This work	5.52	5.60	5.34	5.51
Graphene	PBE	This work	3.76	3.75	3.77	3.74
		2	4.00	3.95	4.02	3.97
	PBE+vdW	This work	3.16	3.18	3.21	3.22
		2	3.29	3.26	3.29	3.27

**TABLE V.** Calculated binding energy (in eV) for DNA nucleobases physisorbed on graphene or Ti_3_C_2_ sheets.

		Reference	A	G	T	C
Ti_3_C_2_	PBE	This work	−0.15	−0.17	−0.11	−0.11
	PBE+vdW	This work	−0.20	−0.19	−0.19	−0.16
Graphene	PBE	This work	−0.10	−0.09	−0.06	−0.07
		2	−0.06	−0.14	−0.08	−0.13
	PBE+vdW	This work	−0.71	−0.74	−0.58	−0.55
		2	−1.00	−1.18	−0.95	−0.93
		31	−0.85	−0.99	−0.76	−0.76

**FIG. 7.**
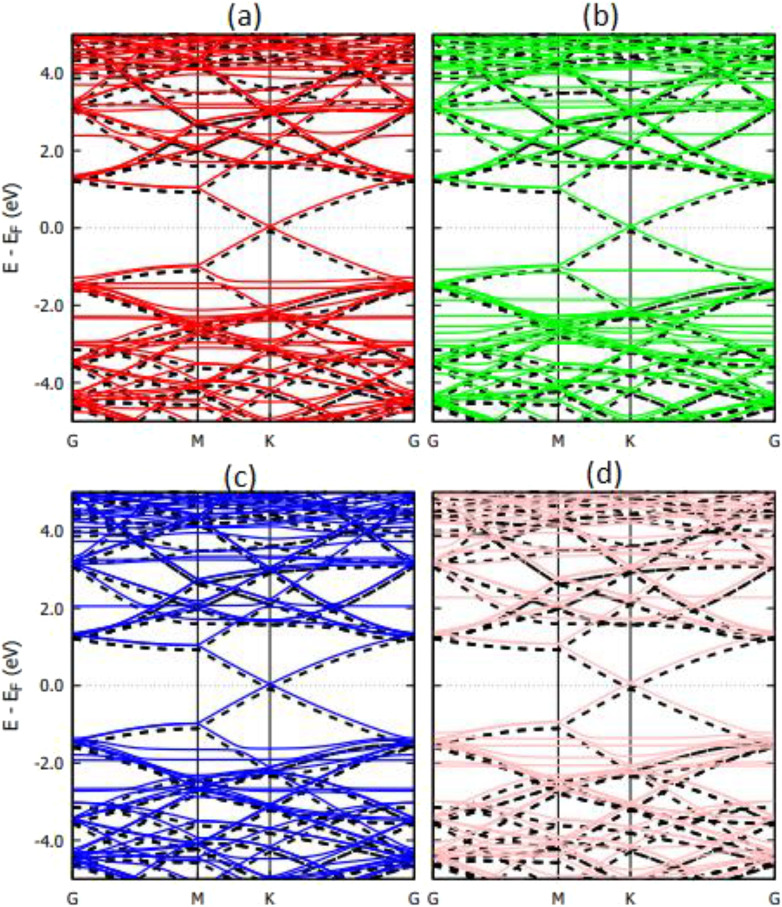
Calculated band structure of graphene with physisorbed DNA nucleobases (a) A, (b) G, (c) T, and (d) C. The dashed black lines represent the band structure of pristine graphene (5 × 5 supercell).

**FIG. 8.**
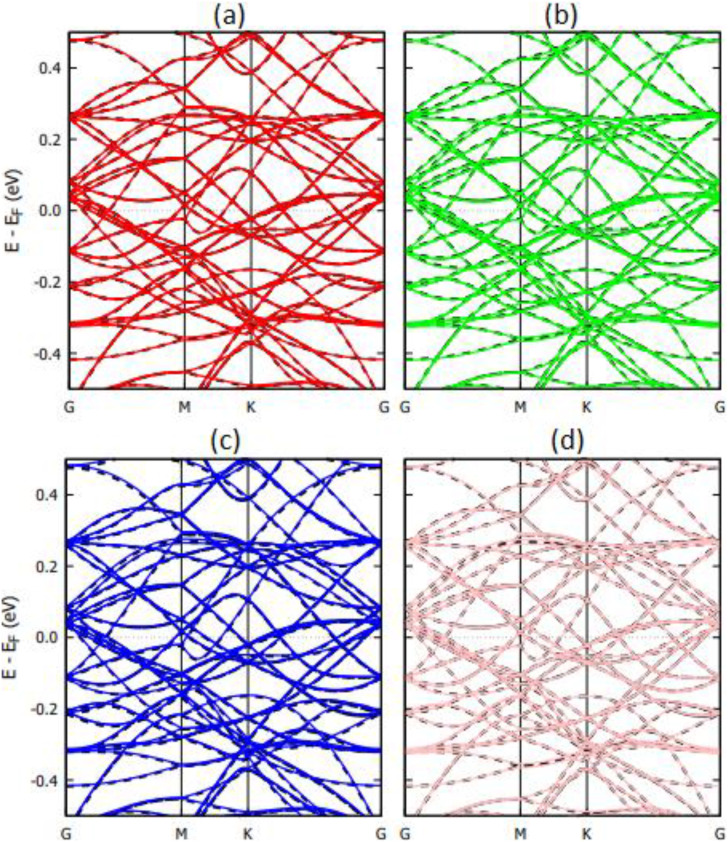
Calculated band structure of Ti_3_C_2_ with physisorbed DNA nucleobases (a) A, (b) G, (c) T, and (d) C. The dashed black lines represent the band structure of pristine Ti_3_C_2_ (5 × 5 supercell).

The band structure of the graphene and Ti_3_C_2_ substrates with DNA bases is shown in Figs. 7 and 8, respectively. In Fig. 7, we observe small perturbations in the band structure of graphene due to the presence of DNA nucleobases. The systems, however, remain gapless and semi-metallic. Small changes in the band structure of a substrate due to physisorption of DNA nucleobases are common and have been reported for the physisorption of nucleobases on graphene and hBN.^2^ Unlike graphene, Fig. 8 shows that the band structure changes of Ti_3_C_2_ upon physisorption of DNA nucleobases are negligible. This correlates with the weak physisorption of nucleobases on Ti_3_C_2_ compared to graphene.^2^

#### 2. Chemisorption of DNA nucleobases on Ti_3_C_2_

**FIG. 9.**
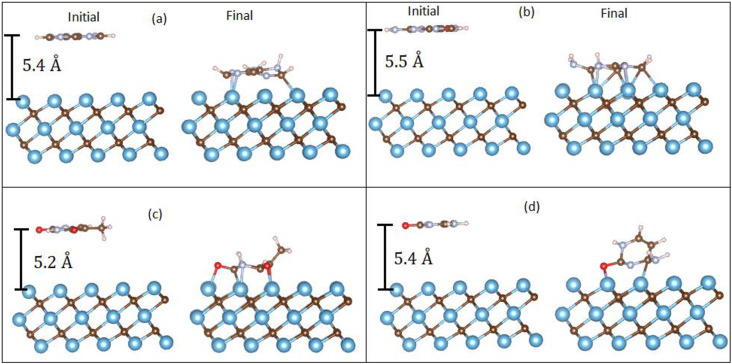
Optimized geometries of Ti_3_C_2_ with chemisorbed DNA nucleobases (a) A, (b) G, (c) T, and (d) C.

As already discussed in Sec. III C, two adsorption mechanisms are possible for DNA nucleobases on Ti_3_C_2_, namely, physisorption and chemisorption. Figure 9 shows the optimized geometries of DNA nucleobases chemisorbed on the Ti_3_C_2_ surface. For chemisorption, A and G chemisorbed with the nucleobases parallel to the surface of Ti_3_C_2_. For T and C, the bases chemisorbed at a tilted angle. The binding energies for chemisorption are shown in Table VI. For the PBE+vdW method, the binding energies follow the order G > A > T > C, which is the same order for the binding energies for physisorption. The binding energy values (5.3–7.5 eV) indicate very strong chemisorption. The chemisorbed binding energies are ∼40 times larger than the physisorbed binding energies (Table V). Such strong chemisorption will cause the DNA nucleobases to stick to the Ti_3_C_2_ and increase the error rates for identification of nucleobases.^14,15^

**TABLE VI.** Binding energy and initial vertical height for chemisorption of DNA bases on Ti_3_C_2_.

Base	PBE		PBE+vdW	
	*d* (Å)	*E*_*b*_ (eV)	*d* (Å)	*E*_*b*_ (eV)
A	4.7	−5.48	5.4	−6.39
G	4.7	−5.89	5.5	−7.49
T	4.1	−3.35	5.2	−6.28
C	4.9	−3.99	5.4	−5.31

**FIG. 10.**
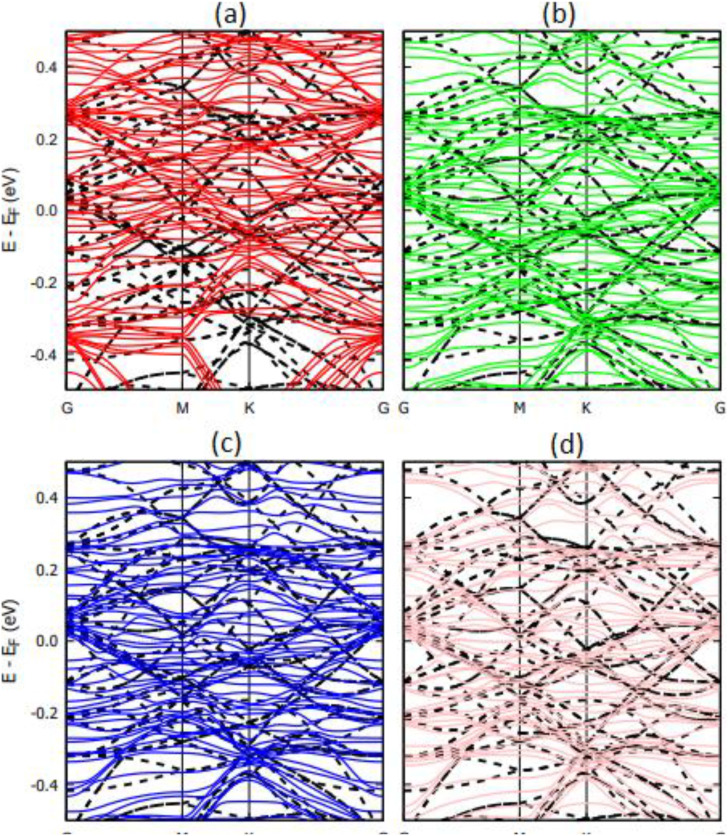
Calculated band structure of Ti_3_C_2_ with chemisorbed DNA nucleobases (a) A, (b) G, (c) T, and (d) C. The dashed black lines represent the band structure of pristine Ti_3_C_2_ (5 × 5 supercell).

The band structures of the Ti_3_C_2_ substrate with DNA bases chemisorbed are shown in Fig. 10. While physisorption of nucleobases on Ti_3_C_2_ produces a negligible change in the band structure (Fig. 8), the chemisorption of nucleobases produces significant perturbations in the band structure.

### D. Electron transport

**FIG. 11.**
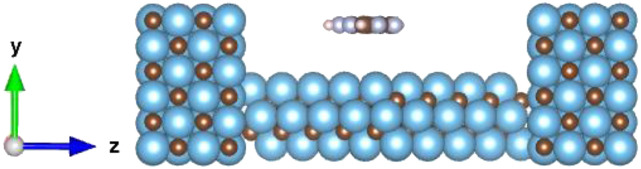
Ti_3_C_2_ device composed of Ti_3_C_2_ (central) and Ti_3_C_2_ electrodes (left and right). The transport direction is the *z* direction.

The electronic transport properties of Ti_3_C_2_ with DNA nucleobases are elucidated by inserting them between semi-infinite left and right electrodes (LE and RE, respectively), forming a device structure. In addition to pristine Ti_3_C_2_ (Ti_3_C_2_, without the base), four other device configurations (given by the physisorption or chemisorption interaction mechanisms) are examined: Ti_3_C_2_+A, Ti_3_C_2_+G, Ti_3_C_2_+T, Ti_3_C_2_+C (Ti_3_C_2_ with a base A, G, T, or C, respectively). To that end, the optimized structures are attached to the Ti_3_C_2_ electrodes, as illustrated in Fig. 11, which presents a representative device structure. In a device, the scattering region (where the system is inserted) contains screening layers of LE and RE as well.^32^ For the relaxation calculation, the force tolerance is set to 0.05 eV/Å, and the electrodes are constrained. The corresponding optimum distance between each electrode surface and the system is associated with the lowest energy configuration. The influence of the nucleobase on the electronic transport characteristics is exhibited through the transmission and device density of states (DDOS). This accompanying analysis is implemented employing the QuantumATK software, which is based on DFT combined with the Non-Equilibrium Green’s Function (NEGF). For the DFT calculations, the exchange-correlation potential is approximated within the generalized gradient approximation (GGA) with the Perdew–Burke–Ernzerhof (PBE) functional (GGA.PBE),^21^ for the exchange and correlation effects of the electrons. PseudoDojo pseudopotentials^33^ are employed for the ion cores. A mesh cut-off energy of 75 hartree with a (1 × 1 × 150) k-point mesh with the Monkhorst–Pack scheme^34^ is utilized. All atoms are identified by their valence electrons whose electronic structures are represented by *medium* basis sets of local numerical orbitals.

**FIG. 12.**
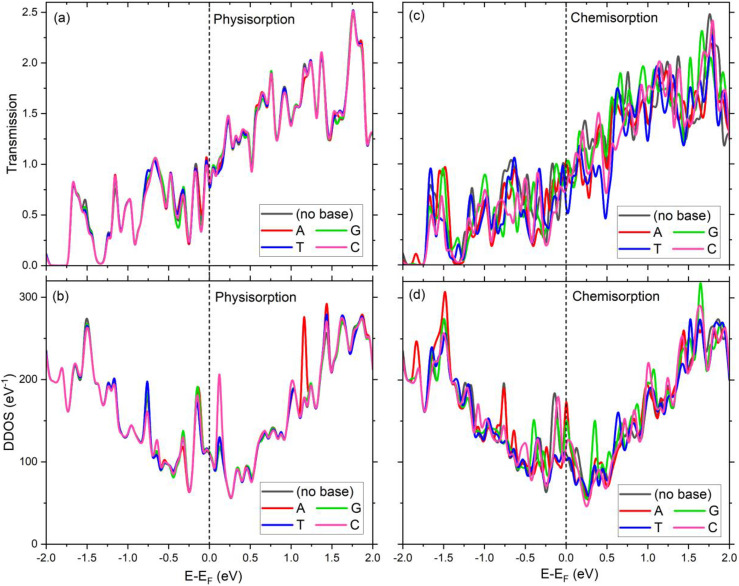
Transmission and device density of states (DDOS) of the Ti_3_C_2_ device structures with and without a base for (a) and (b) physisorption and for (c) and (d) chemisorption interaction mechanisms.

The transmission coefficient and DDOS spectra of each device for both physisorption and chemisorption interaction mechanisms are illustrated in Fig. 12. The electronic transport of a device is determined by the states near the Fermi level (*E*_*F*_, which is set to zero). The transmission is influenced by both electrodes and the electronic structure of a system (i.e., substrate plus base). However, the DDOS is associated with the system attached to them. Hence, a zero transmission at a certain energy may not refer to a zero DDOS at that energy. For all device structures, the transmission shown in Figs. 12(a) and 12(c) is finite at the Fermi energy *E*_*F*_. The nonzero transmission at this specific energy suggests a finite zero-bias conductance. As shown in Fig. 12(a), the transmission due to physisorption displays a dip and is identical for all (0.81) but Ti_3_C_2_+T for which it is slightly lower (0.77). The chemisorption interaction brings about non-identical transmissions at both *E*_*F*_ and other energies, as illustrated in Fig. 12(c). In this case, the transmission of Ti_3_C_2_+T device drops to 0.51, becoming well below that of other devices. The appreciable transmission in the vicinity of *E*_*F*_ suggests that Ti_3_C_2_+ base structures can be employed to develop functional conducting devices. The alteration of transmission with dramatic increments at particular energies is evident. As there is more than one transmission channel in the system, the transmission coefficient can be higher than 1.00. The salient transmission peaks (resonances) shown in Figs. 12(a) and 12(c) reveal the electronic transport characteristics of each device. They refer to the tunneling probability of the electrons across the device. They are governed by system-electrode coupling and electrode surface states.^35^ A finite bias enhances them and results in non-zero electric current.

The available electronic states of the system linked to the electrodes can be revealed with the help of the DDOS, which is shown in Figs. 12(b) and 12(d) for each interaction mechanism. The accompanying electronic properties of Ti_3_C_2_+ base systems are chiefly determined by well-defined and suppressed DDOS peaks. As the states around *E*_*F*_ are crucial in electronic transport, the prominent DDOS peaks right below and above it play a significant role in the electronic nature of Ti_3_C_2_+ base devices. Figures 12(b) and 12(d) depict that the physisorption (chemisorption) mechanism yields nearly identical (non-identical) dramatic increments at certain energy levels. This behavior is also reflected in the corresponding band structure for each mechanism (see Figs. 8 and 10). Due to the physisorption interaction, in addition to roughly identical peaks below *E*_*F*_, a sharp peak representing the Ti_3_C_2_+C device above *E*_*F*_ is obvious [Fig. 9(b)]. Depending on the base, DDOS peaks can deviate from each other at certain energies (such as −0.77, 0.13, and 1.2 eV), as seen in Fig. 12(b). As for the chemisorption interaction, it results in salient DDOS peaks at *E*_*F*_ for both Ti_3_C_2_+A and Ti_3_C_2_+G devices [Fig. 12(d)]. Besides, it also causes noticeable peaks in the vicinity of *E*_*F*_.

## IV. CONCLUSION

In summary, we have investigated the interaction of DNA nucleobases with single-layer Ti_3_C_2_ MXene and graphene using vdW-corrected DFT and NEGF methods. We showed that depending on the initial vertical height of a nucleobase above the Ti_3_C_2_ surface, two interaction mechanisms are possible, namely, physisorption and chemisorption. For graphene, DNA nucleobases always physisorbed onto the graphene surface irrespective of the initial vertical height of the nucleobase above the graphene sheet. The PBE+vdW binding energies for graphene are high (0.55–0.74 eV) and follow the order G > A > T > C, with adsorption heights in the range of 3.16–3.22 Å, indicating strong physisorption. For Ti_3_C_2_, the PBE+vdW binding energies are relatively weaker (0.16–0.20 eV) and follow the order A > G = T > C, with adsorption heights in the range of 5.51–5.60 Å, indicating weak physisorption. The binding energies for chemisorption follow the order G > A > T > C, which is the same order for the binding energies for physisorption. The binding energy values (5.3–7.5 eV) indicate very strong chemisorption. The chemisorbed binding energies are ∼40 times larger than the physisorbed binding energies. Furthermore, our band structure and electronic transport analysis showed that for physisorption, there is neither significant variation in the band structure nor modulation in the transmission function and the device density of states (DDOS). The relatively weak physisorption and strong chemisorption show that Ti_3_C_2_ might not be capable of identifying DNA nucleobases using the physisorption method.

## Data Availability

The data that support the findings of this study are available from the corresponding authors upon reasonable request.
